# Surface Functionalization of Organosilica Nanoparticles With Au Nanoparticles Inhibits Cell Proliferation and Induces Cell Death in 4T1 Mouse Mammary Tumor Cells for DNA and Mitochondrial-Synergized Damage in Radiotherapy

**DOI:** 10.3389/fchem.2022.907642

**Published:** 2022-05-10

**Authors:** Chihiro Mochizuki, Yukihito Kayabe, Junna Nakamura, Masaya Igase, Takuya Mizuno, Michihiro Nakamura

**Affiliations:** ^1^ Department of Organ Anatomy and Nanomedicine, Graduate School of Medicine, Yamaguchi University, Yamaguchi, Japan; ^2^ Core Clusters for Research Initiatives of Yamaguchi University, Yamaguchi, Japan; ^3^ Laboratory of Molecular Diagnostics and Therapeutics, Joint Faculty of Veterinary Medicine, Yamaguchi University, Yamaguchi, Japan

**Keywords:** organosilica nanoparticles, Au nanoparticles, radiotherapy, x-ray sensitizer, imaging assay

## Abstract

Radiotherapy is one of the most effective cancer treatments. Au nanoparticles (NPs) are one of the most used X-ray sensitizing materials however the effective small sub-nm size of Au NPs used for X-ray sensitizers is disadvantageous for cellular uptake. Here, we propose the surface functionalization of organosilica NPs (OS) with Au NPs (OS/Au), which combined the 100 nm size of OS and the sub-nm size of Au NPs, and synthesized effective Au materials as an X-ray sensitizer. The X-ray sensitizing potential for 4T1 mouse mammary tumor cells was revealed using a multifaceted evaluation combined with a fluorescence microscopic cell imaging assay. The number of polyethyleneimine (PEI)-modified OS (OS/PEI) and OS/Au (OS/Au/PEI) uptake per 4T1 mouse mammary tumor cell was the same; however, 4T1 cells treated with OS/Au/PEI exhibited significant inhibition of cell proliferation and increases in cell death by X-ray irradiation at 8Gy. The non-apoptotic death of OS/Au/PEI-treated 4T1 cells was increased by DNA and mitochondrial-synergized damage increase and showed potential applications in radiotherapy.

## 1 Introduction

Radiotherapy is one of the most effective cancer treatments.(Delaney et al., 2005). The common types of tumors have been treated with radiotherapy and combined with surgery, chemotherapy, or immunotherapy. Radiotherapy uses ionizing radiation (IR), which refers to electromagnetic waves such as X-ray and γ ray, and particle radiation such as α, β particles, and neutron beams. (Wang et al., 2018). The mechanism of cell death induced by IR is as follows: The IR interaction with biomolecules causes ionization and excitation to produce reactive oxygen species (ROS) such as hydroxyl radicals (•OH); (Kurzyp et al., 2017; Moriyama et al., 2019); these ROS quickly react with cellular components and damage them. DNA is a primary target for determining the effects of IR on cells. After IR irradiation, the damaged cells are repaired through a series of cellular processes; if this repair is unsuccessful, cell death occurs within seconds to days. (Mah et al., 2010). The selectivity of tumor cells is not sufficiently high in radiotherapy, and exposure to IR can directly damage normal cells and tumor cells. Because of the lower selectivity, the dose of IR has been often limited in clinical applications. (Moding et al., 2013).

A variety of Au nanoparticle (NPs) systems have been tested for their radiosensitization properties by examining their physical properties such as the size, shape, and composition of the surface ligands of NPs *in vitro*. (Her et al., 2017). Several studies have reported the use of 1.9 nm Au NPs (AuroVist™) protected by thiol derivatives. (Rahman et al., 2009; Butterworth et al., 2010; Jain et al., 2011; Coulter 2012; Jain et al., 2014; Taggart et al., 2014). The radiosensitization of several tens of nanometers of Au NPs protected by citrate, (Chang et al., 2008; Chithrani et al., 2010; Liu et al., 2015), glucose, (Kong et al., 2008; Kaur et al., 2013; Wang et al., 2013), and PEG, (Zhang et al., 2012; Chattopadhyay et al., 2013; Joh et al., 2013; Wolfe et al., 2015), and the precise mechanism behind the increase in IR effects attributed to Au NPs is yet to be fully elucidated. One of the most widely believed mechanisms is that of physical enhancement, which is caused by the increased X-ray photoelectric absorption of Au. (Regulla et al., 1998). Precious-metal-enhanced IR is an attractive method in X-ray sensitizers because the precious metal absorption of X-rays is based on the high-Z materials theory that produces secondary electrons (e.g., Auger electrons) which react with intracellular water and generate •OH. (Kuncic and Lacombe 2018; Chen et al., 2019; Tamanoi et al., 2020). Previous studies have reported on the use of small-sized Au NPs (sub-5-nm) as radiosensitizers for producing ROS for the large surface area of Au. (Cheng et al., 2012). This study showed that the turnover frequency (TOF) values, which is the number of catalytic reactions by a surface Au per second, of the hydroxylation of coumarin carboxylic acid for 3 nm Au NPs appeared to be better than that of the 30 nm Au NPs by a factor of 2 for the lowest surface areas. This size dependence was different from that of simple Au NPs catalytic reactions, which increased the number of surface atoms of Au NPs by small Au NPs for the oxidation reaction, (Ishida et al., 2020), indicating chemical enhancement. For these reasons, it is predicted that the X-ray sensitization of Au NPs will be highly effective for the sub-nm size. Au [Z (atomic number) = 79] has been investigated as an X-ray sensitizer owing to its biocompatibility and low cytotoxicity. In the previous study, polymer-supported Au NPs exhibited increased cellular uptake for the DU145 human prostate cancer cell line because of their increased stability under biological conditions compared to that of free Au NPs. (Bennie et al., 2020). Thus, Au NPs supported on stable materials in biological conditions will be highly effective as X-ray sensitizers.

The enhanced permeability and retention (EPR) effect can be utilized to apply Au NPs in radiotherapy based on the drug delivery system (DDS). The EPR effect is attributed to the increased vascular permeability caused by the incomplete neovascularization around the tumor and the lack of excretory function by lymphatic capillaries in the tumor tissues. (Matsumura and Maeda 1986; Maeda et al., 2013). The appropriate NP size for the EPR effect is approximately 5–200 nm; 100 nm NPs are widely used. Thus, the size control of approximately 100 nm NPs is essential for DDS.

Therefore, to achieve more effective Au-enhanced radiotherapy, it is necessary to combine the sub-nm Au NPs (for increased X-ray sensitization) and the 100 nm sized NPs (to target tumor cells). Thus, X-ray sensitizing and targeting tumor cells for DDS, and effective nano-drug for radiotherapy could be achieved by the functionalization of 100 nm NPs with sub-nm sized Au NPs. Among DDS NPs, OS NPs are among the most attractive NPs to realize easy size control to exploit the EPR effect and simple surface functionalization with biomolecules for the preferential accumulation of NPs in the tumor. (Tang and Cheng 2013; Nakamura 2018; Mochizuki et al., 2021).

One of the most common methods used in the evaluation of the effect of IR for the quantitative analysis of IR cell death is a colony-forming assay. (Puck et al., 1957). Cells form single cell colonies when they continue to proliferate. The cells that proliferate and die divide only a few times when they are irradiated; therefore, they do not form large colonies even after culturing for a certain period. However, colony-forming assays require long experimental periods, and therefore, they are not suitable for multiple samples. Water Soluble Tetrazolium Salts (WST-1) and 3-(4,5-dimethylthiazol-2-yl)-2,5-diphenyl-2H-tetrazolium bromide (MTT) assays are commonly used for cell viability assays in IR cell death. (Arab-Bafrani et al., 2016). WST-1 is more convenient than MTT because of its water solubility and storage conditions; however, the WST-1 assay does not observe cell death. IR cell death is characterized by its characteristic morphology, and thus, the multifaceted evaluation of IR cell death *in vitro* needs to be investigated. Fluorescence microscopic cell imaging is a popular method for observing living cells and the structure of organelles with a fluorophore stain; this method can observe cell dynamics in living cells with the fluorophore stain, and its sensitivity allows quantifying the expression of biomolecules such as proteins. A detailed observation of the preserved cells can be realized if cells are fixed to halt cell metabolism before the addition of a fluorophore. (Evanko 2005). Thus, multifaceted evaluation combined with fluorescence microscopic cell imaging can provide efficient tools for IR cell death based on morphology.

We report on the surface functionalization of OS with Au NPs (OS/Au) and evaluate the X-ray sensitizing potential of OS/Au. The OS/Au, which combined sub-nm sized Au NPs and 100 nm sized OS, was synthesized to assess the effect of Au as an X-ray sensitizer. OS/Au showed high •OH producing ability compared to that of OS. Further, we evaluate a mouse mammary tumor cell line as an X-ray sensitizer of 4T1 cells (mouse mammary tumor cell line). The NPs uptake was quantified as the number per 4T1 cells. The uptake number for polymer polyethyleneimine (PEI)-modified OS (OS/PEI) and OS/Au (OS/PEI) were similar, and the effect of Au as an X-ray sensitizer could be evaluated. We also reveal how to damage cells taken up by X-ray irradiation by multifaceted evaluation combined with fluorescence microscopic cell imaging. The OS/Au/PEI-treated 4T1 cells exhibited significantly low cell viability and inhibition of cell proliferation and increases in cell death by X-ray irradiation at 8Gy. The death of OS/Au/PEI-treated 4T1 cells was induced by DNA and mitochondrial-synergized damage. The OS/Au/PEI will provide an effective model for NPs as X-ray sensitizers and the multifaceted evaluation helped us understand cell death in radiotherapy.

## 2 Results and Discussion

### 2.1 Surface Functionalization of Organosilica NPs With Au NPs

**FIGURE 1:**
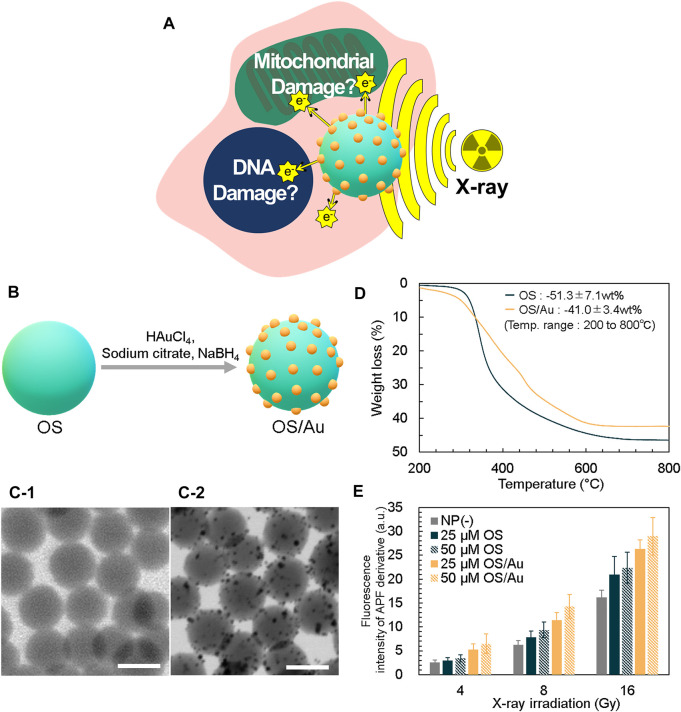
Strategy for X-ray sensitizer and characterization of OS/Au. **(A)** Schematic of the strategy for X-ray sensitizer as cancer cell therapy. **(B)** Detailed overview of the surface functionalization of OS with the Au NPs (OS/Au) of OS/Au. The STEM of OS (C-1) and OS/Au (C-2). Scale bar = 100 nm. **(D)** TGA data of OS and OS/Au. **(E)** Fluorescence intensity of the APF derivative.

Figure 1A shows a schematic of the synergistic radiation enhancement of the surface functionalization of organosilica NPs (OSs) with Au NPs (OS/Au) for DNA and mitochondrial damage. Au NPs have gained considerable research interest as radiosensitizers because of the increased absorption of X-rays and the production of locally damaging surroundings. (Regulla et al., 1998; Cheng et al., 2012; Her et al., 2017). Further, the small size of the Au NPs, which have a large surface area, is more favorable for X-ray sensitizing. (Cheng et al., 2012). However, the stability of electrostatically stabilized Au NPs is an important consideration in biological environments. (Westcott et al., 1998). In our previous reports, OS was synthesized using only MPMS with SH groups as the silica source; therefore, it was easy to control the size of the NPs. (Nakamura and Ishimura 2007; Nakamura et al., 2010). The OS exhibited high stability in a biological environment, and the functionalization of the small Au NPs on OS helped realize the stability of NPs and efficiency of the X-ray sensitizing ability. The targeting capabilities of OS have been improved, and these systems have been applied for labeling tumor cell lines. (Nakamura 2018; Mochizuki et al., 2021). Thus, OS/Au (the combination of OS and Au NPs) are a viable candidate for achieving increased cellular uptake and cell death with radiation enhancement.

We prepared OS containing fluorescein isothiocyanate (FITC) based on our previous report. (Nakamura et al., 2015). The synthesized OS has a spherical shape and narrow size distribution (i.e., mean diameter of 107.0 ± 10.3 nm); further, the OS surface is functionalized with Au NPs, as shown in Figure 1B. First, OS is mixed and stirred with HAuCl_4_ as the Au precursor; the Au and SH groups interact well on the OS surface. Next, NaBH_4_ is added to form Au NPs on the OS surface. The formation of Au NPs with a mean diameter of 4.7 ± 1.6 nm on the OS surface is observed and measured using scanning transmission electron microscopy (STEM) (Figure 1C). The Au content is calculated by thermogravimetric analysis (TGA) (Figure 1D). The profiles of OS and OS/Au obtained in a flow of air exhibit drastic weight loss attributed to the thermal decomposition in air. For OS, the fraction of weight loss in the temperature range of 200–800°C was 51.3% (w/w) (SD = 7.1, *n* = 3); this value is close to the 52.8% (w/w) theoretical weight loss from OS to SiO_2_. The weight loss of OS/Au is 41.0% (w/w) (SD = 3.4, *n* = 3), and the fraction of weight loss by OS/Au is 10.3% (w/w) lower than that of OS. We calculated the Au content of OS/Au as 20.1% (w/w), which is high compared to those in previous reports such as that for the Au NPs composite. This value was in good agreement with that measured by ICP-OES, which was 21.0% (w/w). Since the OS was synthesized using only MPMS with SH groups as the silica source, the abundant SH groups of OS contributed to the functionalization of the high concentration of Au NPs. In addition, the OS has a high specific surface area because its diameter is approximately 100 nm; it is believed to increase the surface area of the Au-NP-functionalized scaffold. Regarding the yields obtained of Au NPs, the traditional Turkevich method (Turkevich et al., 1951) for Au NPs has required a diluted reaction solution (0.25 mM of Au), thus necessitating large quantities of water. The Burst method (Brust et al., 1994) uses a higher concentration of Au (5 mM) but uses water−toluene solvents. Therefore, highly economical and green methods will be needed. This method used 2 mM of Au as the final concentration and the reaction temperature was 37°C. The large-scale synthesis would be applied for application study *in vivo* and clinical use.

The extinction spectra of OS/Au shows new peaks at 527 nm compared to those of OS, which is a good assignment to the surface plasmon resonance (SPR) band of the silica-supported Au NPs (Supplementary Figure S1). The fluorescence peak of OS excited at 488 nm was observed at approximately 530 nm; it originated from the fluorescence of FITC in OS. The fluorescence of OS/Au exhibited a marked decrease caused by absorption because of the formation of Au NPs on the surface of the OS (Supplementary Figure S1). The fluorescence signal of OS/Au was detected by fluorescence microscopy (FM) imaging.

The radiolysis of water generates ROS, i.e., •OH. We evaluated the X-ray sensitizing ability of OS and OS/Au by measuring the •OH production. Further, one of the primary pathways of IR cell death indirectly causes cellular damage by inducing oxidative stress. (Vignard et al., 2013). The quantity of •OH is measured using the fluorescence intensity of an aminophenyl fluorescein (APF) derivative, which interacts with •OH and APF (Figure 1E). (Nakayama et al., 2016; Moriyama et al., 2019) The fluorescence intensity of the APF derivative increases from the NP (-) condition. Initially, we investigated the effect of the NP concentration at 4 Gy irradiation, and the fluorescence intensity of the APF derivative for OS and OS/Au increased from1.15 to 1.32 and 2.01 to 2.49-fold, respectively, compared to that in the absence of NPs (NP (-)) because the NP concentration increased from 25 to 50 μg mL^−1^. At 16 Gy irradiation, the fluorescence intensity of the APF derivative of OS and OS/Au increased from 1.30 to 1.38 and 1.62 to 1.79-fold, respectively, compared to the fluorescence intensity exhibited in NP (-) because the NP concentration increased from 25 to 50 μg mL^−1^. The increase in the fluorescence intensity of the APF derivative indicated its dose-dependent effect on OS and OS/Au.

Subsequently, we investigated the dose-dependent effects of X-ray irradiation. In NP (-), the FL intensity of APF increased 2.40 and 6.24-fold as X-ray irradiation dose increased from 4 to 8 and 16 Gy, respectively. For 25 μg mL^−1^ of OS, the fluorescence intensities of the APF derivatives increased to 2.98, 7.82, and 20.96 as the X-ray irradiation dose increased from 4 to 8 Gy and then to 16 Gy, respectively. For 25 μg ml^−1^of OS/Au, the fluorescence intensities of the APF derivatives increased to 5.21, 11.39, and 26.33 as the X-ray irradiation doses increased from 4 to 8 and to 16 Gy, respectively. For 50 μg mL^−1^ of OS and OS/Au, the fluorescence intensities of the APF derivatives exhibited an X-ray dose-dependent behavior. These results indicate that both OS/Au and OS exhibited X-ray sensitizing properties. The hydroxyl radical-generating ability of OS/Au appeared to be stronger than that of OS.

The cellular uptake of NPs is an important factor for achieving highly efficient nanodrugs. (van der Meel et al., 2019). NPs can passively uptake macrophages via endocytosis. For macrophages, NPs can be applied for superior active targeting by functionalization with antibodies binding on their surface, which is based on the interaction between the antibodies and receptors on cells. As another active targeting approach, the positively charged NPs need to be taken up to the negatively charged cell surface through electrostatic interaction with the negatively charged cell surface. (Nakamura 2018). In this study, OS was easily functionalized with the positively charged polymer PEI. This PEI model is applied as the positive control for cellular uptake and is efficient for evaluating NPs as a drug using cells isolated *in vitro*. (Zimmermann et al., 2006). Therefore, we modified OS/Au with PEI to accelerate the cellular uptake of NPs by 4T1 cells.

The results of the dynamic light scattering (DLS) measurements are presented in Table 1. The distribution of the diameters is shown in Supplementary Figure S2. For the hydrodynamic diameter in water, the diameter of the OS was found to be larger than that measured by STEM; OS/PEI exhibited a larger diameter than OS. These results are similar to those reported previously. (Doura et al., 2018). OS/Au exhibited a slightly larger hydrodynamic diameter of 136 ± 11 nm than that of an OS of 132 ± 7 nm; OS/Au/PEI had a larger diameter (145 ± 10 nm) than that of OS/Au. The surface functionalization of OS with Au NPs had a negligible effect on the hydrodynamic diameter compared with that of PEI. The zeta potential of the OS was negative because of the thiol and silanol residues present on the surface. OS/PEI exhibited a positive zeta potential because of the functionalization of OS with the intramolecular amino group moiety of PEI on their surface. For the surface functionalization of OS with Au NPs, OS/Au exhibited negative zeta potentials. The surface of the thiol residue, which interacted with the Au NPs, was removed from the OS by the Au NPs. The surface-functionalized Au NPs on OS were capped with the carbonyl group, which originated from sodium citrate as a stabilizer for the Au NPs. Thus, the negative zeta potential of OS/Au was attributed to the carbonyl and silanol residues. OS/Au/PEI exhibited a positive zeta potential (9.1) upon the functionalization of OS/Au with PEI; however, this value is smaller than that of OS/PEI (36.2). These results suggest that the interaction of PEI and OS/Au is weaker than that of OS, which indicates that the interaction of PEI with the carbonyl group is weaker than that with the thiol group. For the NPs in Dulbecco’s modified Eagle’s medium containing 10% fetal bovine serum and antibiotics (DMEM (+/+)), the hydrodynamic diameter of all NPs was larger than that in water. The zeta potential of all NPs showed a negative charge. These results suggested the adsorption of protein of DMEM (+/+). Notably, the diameter of OS/Au/PEI in DMEM (+/+) was quite large, meaning the interaction with protein was strong.

**TABLE 1:** Hydrodynamic diameters and zeta potentials of NPs by DLS measurement and the evaluation of cellular uptake of NPs by FCM and ICP-OES measurements.

	DLS^a^			DLS^b^			FCM		ICP-OES	
	Hydrodynamic Diameter (nm)	PD Index	Zeta Potential (mV)	Hydrodynamic Diameter (nm)	PD Index	Zeta Potential (mV)	Uptake Population	Uptake Level	NPs Wight^c^/Cell (pg)	NPs Number/Cell (Number)
							(%)	(F.I.)		
OS	132 ± 7	0.040	−36	158 ± 13	0.134	−7	2.5 ± 1.6	25.1 ± 2.0	1.05	875
OS/PEI	143 ± 14	0.127	36	194 ± 18	0.180	−8	96.9 ± 1.9	381.8 ± 120.7	0.88	733
OS/Au	136 ± 11	0.065	−28	148 ± 10	0.101	−5	21.9 ± 8.0	21.3 ± 0.9	0.22	181
OS/Au/PEI	145 ± 10	0.107	9	330 ± 0	0.000	−9	84.1 ± 8.9	96.0 ± 17.3	0.90	750

a NPs were dispersed in water solution.

b NPs were dispersed in DMEM (+/+) medium.

c The quantities of up-taken NPs per particle uptake-positive cell determined from FCM uptake rate

### 2.2 Quantification of the Cellular Uptake of Nanoparticles

**FIGURE 2:**
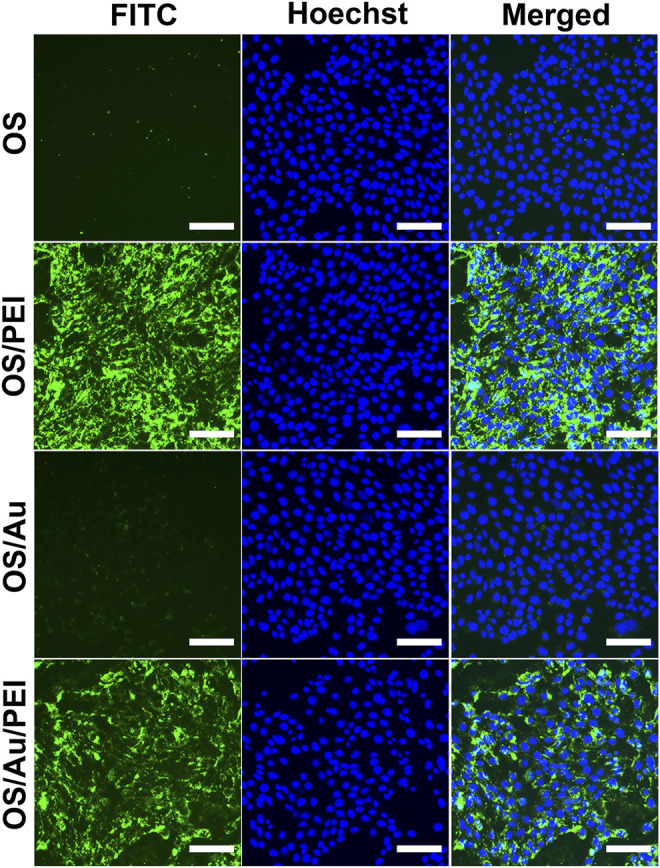
Fluorescence microscopic observation of the cellular uptake of NPs. The cells treated with OS, OS/PEI, OS/Au, and OS/Au/PEI were observed by fluorescence microscopy. Scale bar = 100 μm.

The efficiency of the cellular uptake of NPs is evaluated using FM and flow cytometry (FCM) analysis, as shown in Figure 2. The 4T1 cells (a mouse mammary tumor cell line) were treated with OS, OS/PEI, OS/Au, and OS/Au/PEI (The characterizations of NPs were described in Table 1). The FM observations indicated that the OS uptake was not observed in 4T1 cells. In contrast, positively charged OS/PEI and OS/Au/PEI were observed in almost all 4T1 cells. A small quantity of OS/Au was observed because of the low intensity of the fluorescent signal of OS/Au in a few 4T1 cells.

Next, we performed FCM to evaluate the cellular uptake efficacy; the evaluated values are summarized in Table 1. The cellular uptake efficacy was evaluated using the uptake population (uptake cells per all cells (%)) and uptake level fluorescence intensity of the uptake cells. Cells with a geometric mean above 14 on FL1 were defined as uptake cells; the uptake intensity was calculated based on the geometric mean of the uptake cells. As summarized in Table 1, the uptake of OS by the 4T1 cells was low; they exhibited 96.1 and 84.1% higher uptake ratios of OS/PEI and OS/Au/PEI, respectively. The evaluation of the fluorescence intensity was not discussed because of the difference in the fluorescence intensities of the OS and OS/Au. Further, for OS/Au (without PEI), 27% of the 4T1 cells took up OS/Au. The uptake fluorescence intensity of OS/Au and OS/Au/PEI for the uptake cells was dissimilar; the cellular uptake fluorescence intensity of OS/Au/PEI was 4.1-fold higher than that of OS/Au alone.

The cellular uptake of NPs by 4T1 cells was quantified using inductively coupled plasma optical emission spectroscopy (ICP-OES). The quantity of NPs taken up (pg cell^−1^) was calculated from the results of ICP-OES; the number of cells was counted before cell lysis, and the uptake ratio was determined by flow cytometry. The number of NPs taken up was calculated from the results of the quantities of NPs taken up (pg cell^−1^) and the value (g NPs^−1^ (density: 2.3)) of the single NPs. The OS uptake to the cells was 875 per single cell; however, the uptake population of OS was only 3% according to the results of FCM. A total of 733 OS/PEI and 750 OS/Au/PEI were used up to 4T1. The PEI modification of OS and OS/Au markedly enhanced the uptake of NPs for both OS and OS/Au. Furthermore, although the number of OS/Au taken up by the 4T1 cells was lower than that of OS/Au/PEI, 181 OS/Au were taken up by the 4T1 cells. The uptake of OS/Au/PEI was 4.5-fold higher than that of OS/Au; this result was in good agreement with the FCM results. These results indicate that PEI modification accelerates the cellular uptake of 4T1 cells, and Au functionalization promotes the uptake by 4T1 cells compared to that by the naked OS. This detailed quantification of the number of NPs clearly showed that the cellular uptake ability of OS/PEI and OS/Au/PEI was comparable. Thus, it is possible to evaluate the X-ray sensitizing effect of Au modification on OS/PEI and OS/Au/PEI and provided the effect of the functionalized Au NPs excluded the other factor such as size, surface ligand, or up-taken number of NPs.

### 2.3 Cell Viability of 4T1 Cells With X-Ray Irradiation *in vitro*

We investigated the viability of the 4T1 cells in the presence of NPs with X-ray irradiation using a combination of the imaging assay of the occupied nucleus area and WST-1 assay; the assays were conducted in parallel. The advantages of the imaging assay of the occupied nucleus area are:

(1) the occupied nucleus area refers to the number of cells and,
(2) the types of death by X-ray irradiation are morphologically recognized from the nucleus shape of the structure.

The WST-1 assay is commonly used to evaluate cell viability as a measure of mitochondrial activity.

**FIGURE 3:**
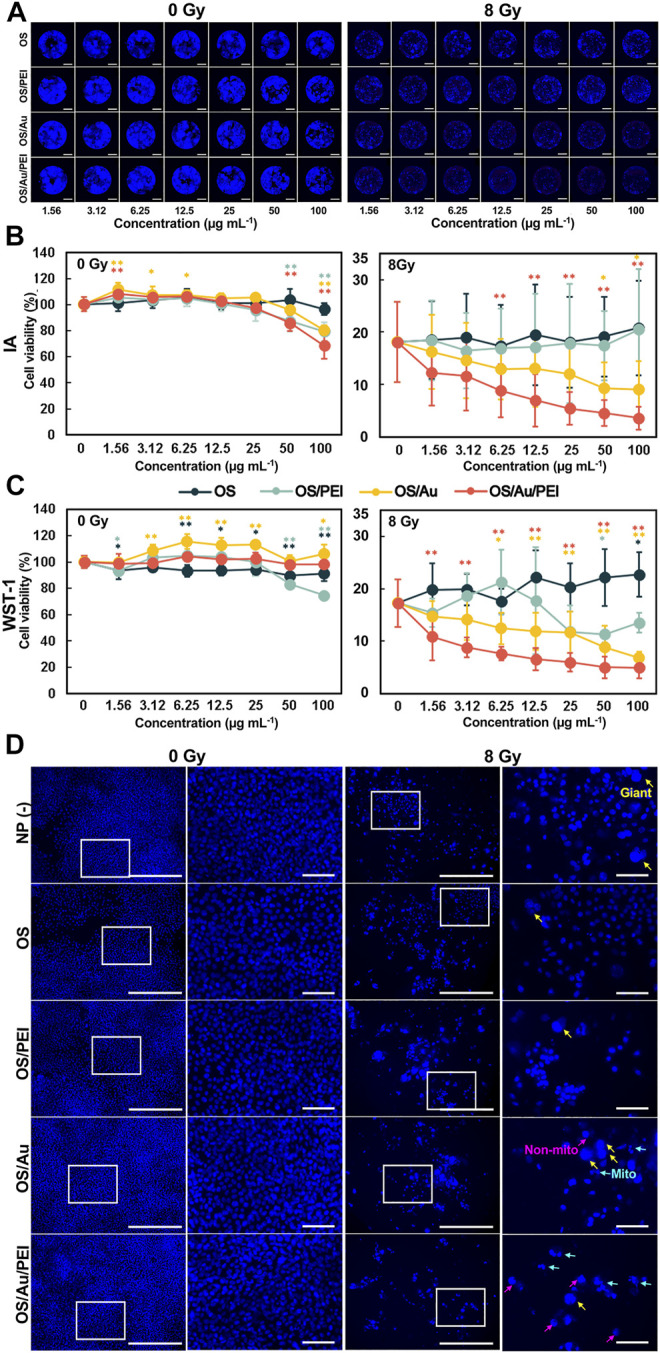
Cell viabilities for 4T1 cells treated with various concentrations of OS, OS/PEI, OS/Au, and OS/Au/PEI as determined via the IA [**(A)**; representative imaging of FM for IA assay (Scale bar = 2 mm), and **(B)**] and WST-1 assays **(C)**. Each value represents mean ± SD, where *n* = 9 (three replicates for three independent experiments). * Significantly different from the 4T1 cells treated with 0 μg mL^−1^ of NPs. **(D)** FM images [left (Scale bar = 500 μm)] and high-resolution FM images [right (Scale bar = 500 μm)] of 4T1 cells. The 4T1 cells were treated with 10 μg mL^−1^ of NP (-), OS, OS/PEI, OS/Au/PEI, and 0 and 8 Gy, respectively. Images of blue fluorescence from Hoechst 33342 staining.

Figure 3B illustrates the results of the imaging assay calculated from Figure 3A. For the imaging assay at 0 Gy, none of the NPs (OS, OS/PEI, OS/Au, OS/Au/PEI) exhibited cytotoxicity up to 25 μg ml^−1^. For OS/PEI, OS/Au, and OS/Au/PEI marginally affected cell viability at concentrations greater than 50 μg ml^−1^.

The cell viability decreased markedly to 18%, even without NP treatment, with X-ray irradiation (8 Gy). Furthermore, OS/Au and OS/Au/PEI caused dose-dependent cell death at the NP concentrations. OS/Au/PEI significantly exhibited cytotoxicity (8.8% cell viability, a 52% decrease compared with the cytotoxicity without NPs) even at the 6.25 μg mL^−1^ concentration of the NPs. In contrast, OS and OS/PEI did not induce dose-dependent cell death at the NP concentrations.

From Figure 3C, it is evident that the results of the WST-1 assay without X-ray irradiation (0 Gy) of all NPs (OS, OS/PEI, OS/Au, OS/Au/PEI) did not exhibit cytotoxicity up to 25 μg mL^−1^. Even at concentrations of up to 100 μg mL^−1^, no cytotoxicity was observed except for OS/PEI. For OS/PEI, the high concentrations of 50 and 100 μg mL^−1^ resulted in a slight decrease in cell viability; OS/Au can activate 4T1 cells. The results of the WST-1 assay exhibited a higher cell viability for OS/Au/PEI compared to the results of the imaging assay. The occupied nucleus area could only be inferred from the adhered cells in the well, and therefore, the non-adhered cells could not be counted. Adherent cells float as they become weaker; therefore, the results of the imaging assay from the occupied nuclear area were lower than the results obtained from the WST-1 assay.

The cell viability from the WST-1 assay showed good agreement with the results of the imaging assay with the X-ray irradiation (8 Gy). The cell viability decreased noticeably to 18% even without NP treatment. Furthermore, OS/Au and OS/Au/PEI caused dose-dependent cell deaths at NP concentrations. For OS/Au, cytotoxicity was gradually observed at a concentration of 6.25 μg ml^−1^ of NPs. The OS/Au/PEI significantly exhibited cytotoxicity (11% cell viability, a 38% decrease compared with that of the cytotoxicity without NPs) even for a concentration of 1.56 μg ml^−1^ of the NPs. The cell viability at 12.5 and 100 μg ml^−1^ of OS/Au/PEI were 7 and 5%, respectively; this means that cytotoxicity was observed at 12.5 μg ml^−1^ and at the high concentration of 100 μg ml^−1^ of OS/Au/PEI. In contrast, OS and OS/PEI did not induce dose-dependent cell death at the NP concentrations. The sensitivity of the detectable WST-1 assay was higher than that of the imaging assay with an 8 Gy irradiation. In the imaging assay, the cell viability would become larger than that in the WST-1 assay because some of the cells of the nucleus became larger after the 8 Gy irradiation. (Cho et al., 2004).

The morphology of the nuclei in the imaging assay (IA) assay wells was observed (Figure 3D). The process of radiation-induced cell death in cancer cells can be classified into mitotic and non-mitotic deaths in terms of the cell cycle. (Forrester et al., 1999). Mitotic death is a state in which the cell loses its ability to proliferate or death occurs through cell division; the morphological characteristics of the nucleus are split nucleus, fusion, or macronucleus. The death of a non-proliferating cell that occurs during interphase without division is defined as non-mitotic death; the morphological characteristics of the nucleus are observed as a rounded shape instead of the oval shape of the normal nuclei.

At 0 Gy, the morphology of the nuclei was similar to that of a normal cell, with flat oval nuclei fluorescing uniformly blue under all conditions, with or without NP. At 8 Gy, we focused on normal nuclei observed in NP (-), OS, and OS/PEI; normal nuclear morphology is rare in PEI. Mitotic (mito-nuclei) and non-mitotic nuclei (non-mitotic nuclei) were frequently observed in OS/Au and OS/Au/PEI, more especially in OS/Au/PEI. (Kobayashi et al., 2017). The high doses of IR are required for non-mitotic death. From the morphology of the nuclei, 4T1 cells treated with OS/Au/PEI exhibited damaged nuclei structure and high sensitivity to X-ray irradiation; the OS/Au/PEI uptake by the 4T1 cells provides selective radiosensitivity by controlling the NP uptake.

The results of the IA and WST-1 assays showed a similar trend, although the sensitivity of the WST-1 assay was marginally higher. In addition, the IA assay was calculated from the occupied nucleus area of the full wells, and the results reflect all populations of the full wells. Furthermore, IA was not limited to evaluating cell viability but could also observe morphological changes in cells. The IA assay has the potential to be developed with more multifaceted information through further detailed analysis because marked morphological changes could be observed by cell death with X-ray irradiation. In addition, the IA assay can be performed in parallel with the WST-1 assay. This combined assay will allow for a more detailed evaluation of the X-ray sensitizing ability.

### 2.4 Inhibition of Cell Proliferation and Increases in Cell Death by OS/Au/PEI With X-Ray Irradiation

**FIGURE 4:**
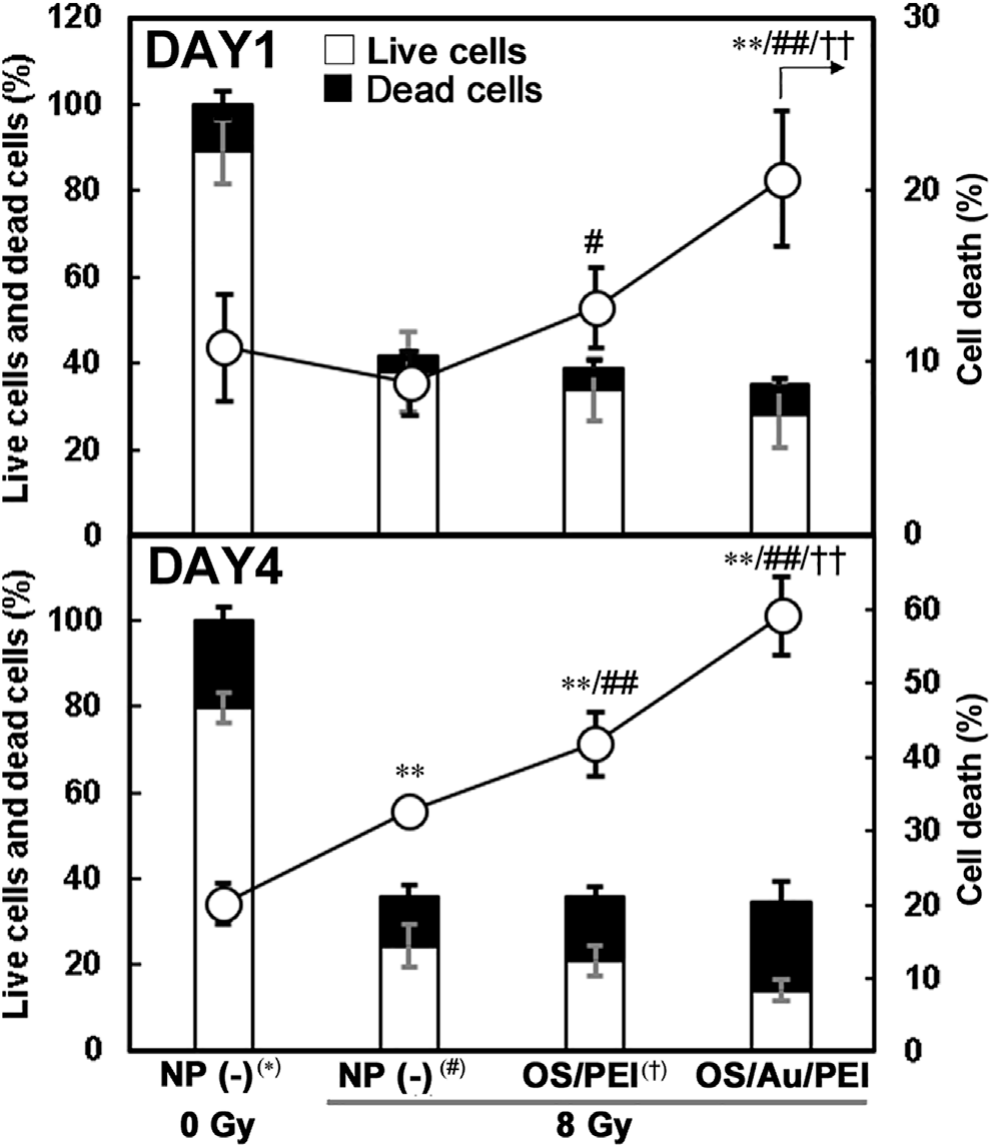
Cell counts and cell viability of 4T1 cells treated with 30 μg ml^−1^ of NP (-), OS, OS/PEI, and OS/Au/PEI with X-ray irradiation. * Significantly different from the 4T1 cells treated with 0 Gy NP (-). # Significantly different from the 4T1 cells treated with 8 Gy NP (-). † Significantly different from the 4T1 cells treated with 8 Gy OS/PEI.

Cell counts and cell viability counts obtained using the trypan blue method clearly showed the effect of X-rays during the very early stages (Figure 4); at DAY1 after X-ray irradiation, approximately 40% of the cells were counted by X-ray irradiation with or without NP treatment. In addition, for the 8 Gy irradiation, the cell death ratio of those cells treated with OS/Au/PEI was 20% and significantly higher than those treated with NP (-) and OS/PEI, even at DAY1 after X-ray irradiation. At DAY4 after X-ray irradiation, less than 40% of the cells were counted by X-ray irradiation with or without NP treatment. In addition, the cell death ratio under all conditions was higher than that for DAY1. These results indicated that X-ray irradiation suppressed cell division. In addition, OS/PEI and OS/Au/PEI induced an increase in the rate of cell death as X-ray sensitizers. The number of live cells treated with OS/Au/PEI was significantly lower than that of cells treated with OS/PEI. A low live cell count is expected to be useful in radiotherapy. Liu et al. reported that tridecapeptide-capped Au_25_ clusters increased ROS levels and inhibited of thioredoxin reductase 1 (TrxR1) activity in cells. TrxR1 is involved in cell viability and proliferation as the regulator of the cellular redox environment in mammalian cells. (Liu et al., 2014). However, the precise mechanisms are not elucidated for leading to the biological enhancement of radiation effect. The evaluation of cell death by X-irradiation by conventional methods is time-consuming, (Puck et al., 1957), however, the evaluation of cell death by cell counting showed a significant increase in cell death in OS/Au/PEI as early as DAY 1 after X-irradiation. Cell counting, which can evaluate cytotoxic activity at an early stage, is a useful evaluation method.

### 2.5 DNA Damage by OS/Au/PEI With X-Ray Irradiation

IR can cause direct and indirect DNA damage, including by the radiolysis of the surrounding water and the generation of a cluster of •OH. In a previous report, indirect DNA damage from •OH accounted for approximately 65% of the radiation-induced DNA damage. (Vignard et al., 2013). DNA double-strand breaks (DSBs) are considered the most lethal among the various types of DNA damage. In the early stages of the cellular DSBs, the histone H2A variant, H2AX, is phosphorylated at the site of DNA damage, which gives rise γ-H2AX (phosphorylated H2AX) at the site of the DNA damage. Thus, we evaluated DNA damage by immunofluorescence staining for γ-H2AX.

**FIGURE 5:**
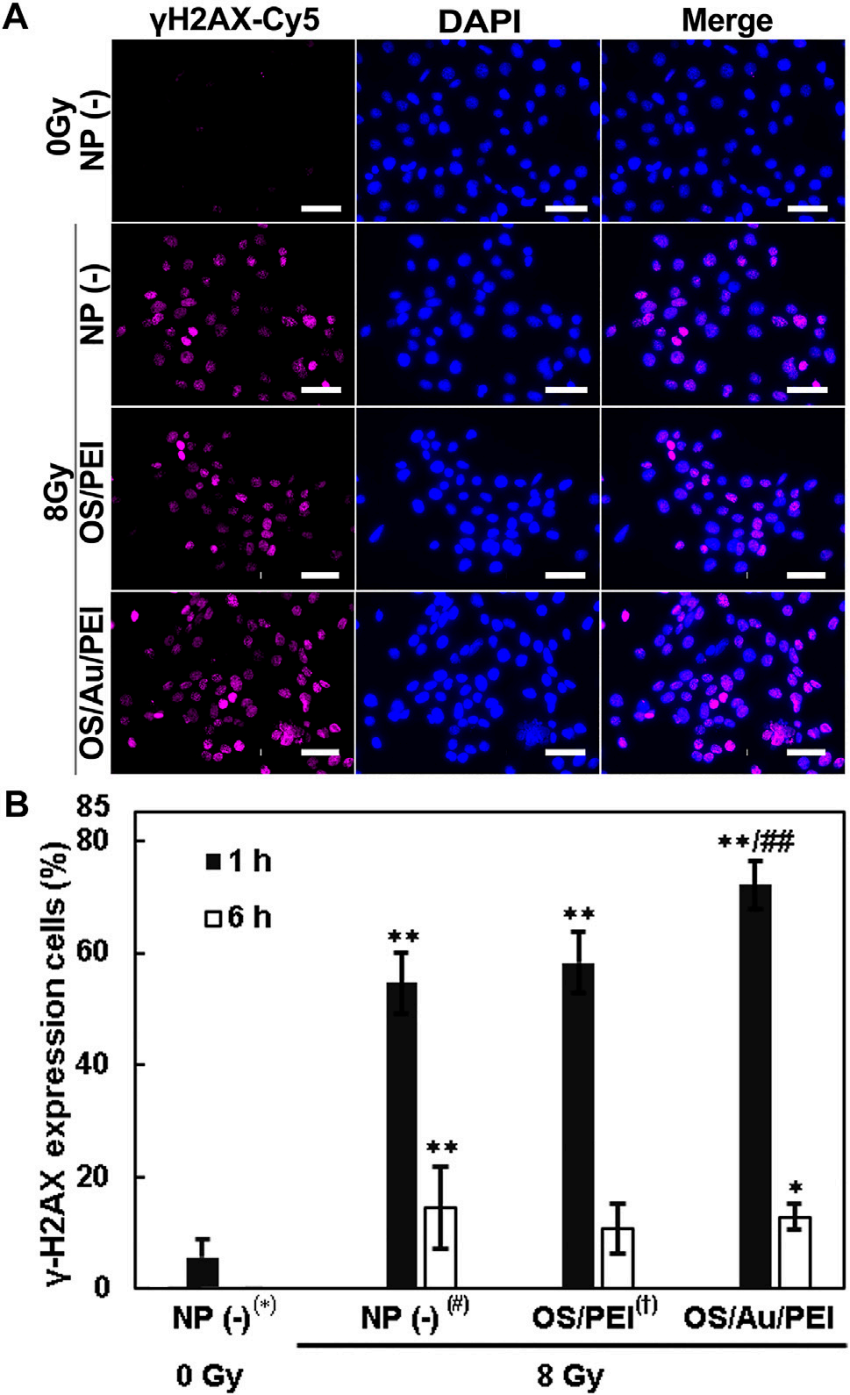
**(A)** FM images of 4T1 cells stained blue with DAPI (indicates the cell nucleus) and stained magenta with Cy5 (indicates the γ-H2AX) treated with 30 μg ml^−1^ of NP (-), OS, OS/PEI, and OS/Au/PEI with X-ray irradiation after 1 h. Scale bar = 50 μm. **(B)** γ-H2AX expression in 4T1 cells treated with 30 μg ml^−1^ of NP (-), OS, OS/PEI, and OS/Au/PEI with X-ray irradiation after 1 and 6 h * Significantly different from the 4T1 cells treated with 0 Gy NP (-). # Significantly different from the 4T1 cells treated with 8 Gy NP (-). † Significantly different from the 4T1 cells treated with 8 Gy OS/PEI.

Figure 5A illustrates the FM images of the 4T1 cells in which the cell nuclei that stained blue with DAPI and γ-H2AX are viewed as magenta Cy5 fluorescence after 1 h of X-ray irradiation. For untreated NP (-) cells (treated with 0 Gy as the negative control), little γ-H2AX was observed in the cell nuclei. However, a magenta Cy5 fluorescence of γ-H2AX was observed in the nucleus in NP (-), OS/PEI, and OS/Au/PEI for cells treated with 8 Gy X-ray irradiation. In addition, the area of the magenta Cy5 fluorescence of γ-H2AX decreased in the order NP (-) < OS/PEI < OS/Au/PEI.

The percentage of stained γ-H2AX cells per cell is calculated as shown in Figure 5B. NP (-) treated with 0 Gy, 6% cells showed γ-H2AX positive. The cells treated with NP (-), OS/PEI, and OS/Au/PEI showed 54, 58, and 72% γ-H2AX expression, respectively, after 1 h of 8 Gy X-ray irradiation. This implies that 8 Gy X-ray irradiation induced DNA damage, and the DNA damage increased with NP treatment. The rate of DNA damage increased with increasing •OH production (Figure 1E). The repair of DSBs is essential for maintaining genomic stability in all cells. (Vignard et al., 2013; Qin et al., 2015). Previous studies reported no influence of AuNPs on the DNA repair kinetics while some studies demonstrated the inhibition of radiation-induced DNA damage repair with AuNPs. (Her et al., 2017). The involvement of Au NPs in the inhibition of DNA repair remains inconclusive. We evaluated the γ-H2AX expression in the cells 6 h after 8 Gy X-ray irradiation. The γ-H2AX expression in cells treated with NP (-), OS/PEI, and OS/Au/PEI was 14, 11, and 13%, respectively. The γ-H2AX expression in NP (-) and OS/Au/PEI cells was slightly higher than that in NP (-) cells treated with 0 Gy as the negative control. The ability to repair DNA damage did not differ; however, the rate of cell death (Figure 4) reflects the result of the DNA damage effected. Therefore, DNA damage caused by X-ray irradiation and NP treatment affects cell survival.

### 2.6 Deactivated Mitochondrial Activity by OS/Au/PEI

Previous studies demonstrated that AuNPs induced ROS formation. (Pan et al., 2009; Li et al., 2010). Further, the resulting oxidative stress has been discussed as an important mechanism of inorganic and metallic NPs-induced cytotoxicity. (Nel et al., 2006). In vitro systems, the AuNP-induced elevation of intracellular ROS levels elicits a multitude of biological effects that ultimately lead to cell death. The cellular response of 1.4 nm triphenylphosphine monosulfonate-capped AuNPs (Au1.4MS) was reported; the AuNPs treatment was associated with the increased ROS production and loss of the mitochondrial potential, which resulted in necrotic cell death. (Pan et al., 2009). The mechanism of AuNPs triggered ROS generation remains uncertain. However, it is believed to occur through the impairment of mitochondrial function as a consequence of the elevated intracellular ROS. (Taggart et al., 2014; Ghita et al., 2017). Therefore, we evaluated the mitochondrial activity using the mitochondrial membrane potential by tetramethylrhodamine methyl ester (TMRE) staining. Positively charged TMRE readily accumulates in active mitochondria because of its relatively negative charge. In contrast, depolarized or inactive mitochondria exhibit decreased membrane potential and fail to sequester TMRE.

**FIGURE 6:**
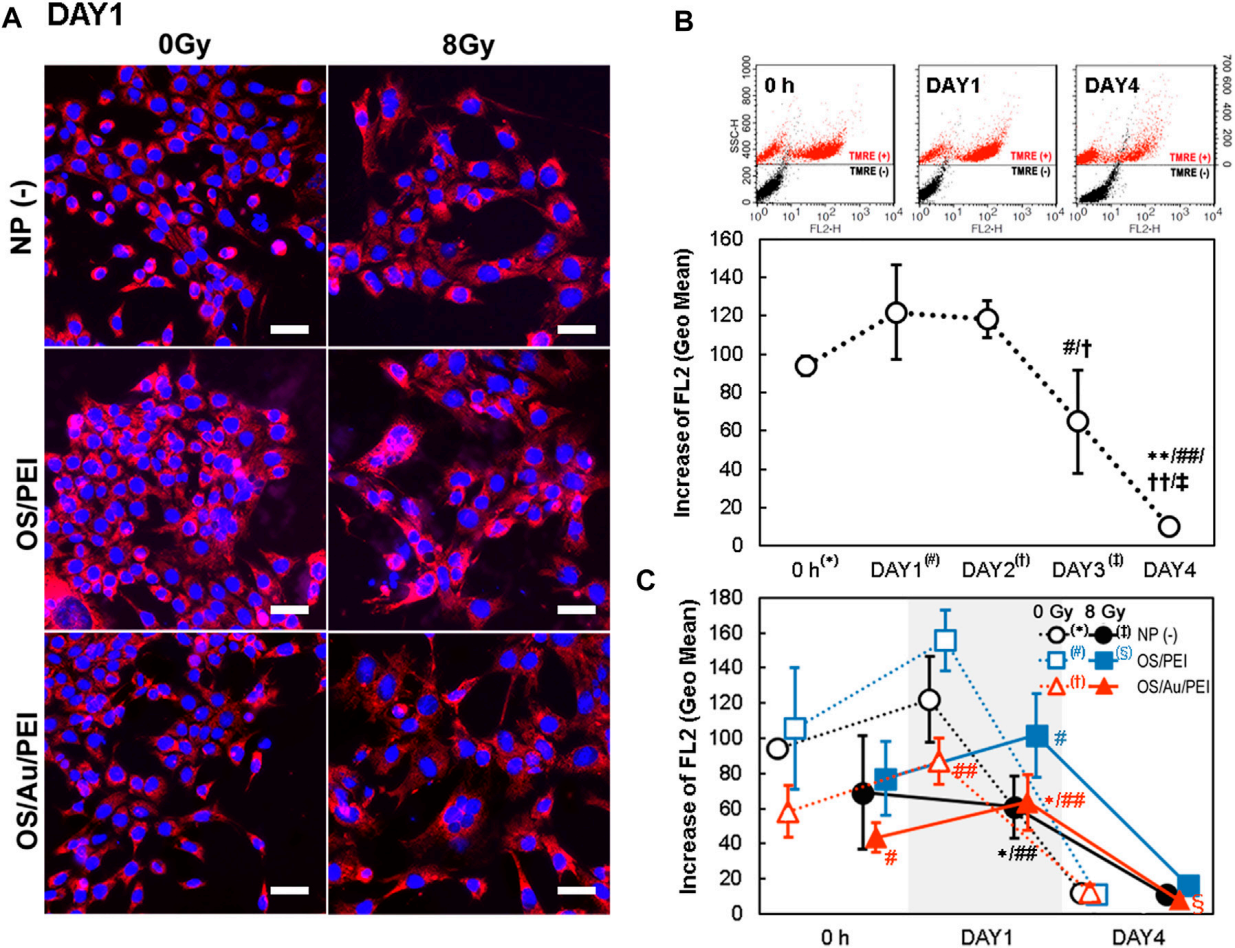
**(A)** TMRE stained FM images of 4T1 cells at DAY1 after X-ray irradiation. 4T1 cells were treated with NP (-), OS, OS/PEI, OS/Au/PEI, and 0 and 8 Gy, respectively. Scale bar = 50 μm. **(B)** Time-dependence of flow cytometric analysis of 4T1 cells with NP (-) and stained with TMRE. (Upper) The SSC values of cells stained with and without TMRE are presented on the left and right sides, respectively. (Lower) Increases in FL2 were calculated by subtracting the fluorescence intensity (geo mean) of cells treated with NPs and stained with TMRE from the control cells treated with NPs but not stained with TMRE. Each value represents mean SD, where *n* = 3 replicates. * Significantly different from the 4T1 cells at 0 h # Significantly different from the 4T1 cells at DAY1. † Significantly different from the 4T1 cells at DAY2. ‡ Significantly different from the 4T1 cells at DAY3. **(C)** Time-dependence of flow cytometric analysis of 4T1 cells with NP (-), OS/PEI, and OS/Au/PEI with or without X-ray irradiation, and stained with TMRE. * Significantly different from the 4T1 cells treated with 0 Gy NP (-). # Significantly different from the 4T1 cells treated with 0 Gy OS/PEI. † Significantly different from the 4T1 cells treated with 0 Gy OS/Au/PEI. ‡ Significantly different from the 4T1 cells treated with 8 Gy NP (-). § Significantly different from the 4T1 cells treated with 8 Gy OS/PEI.

Figure 6A illustrates the FM images of the 4T1 cells of NP (-), OS/PEI, and OS/Au/PEI-treated with 0 and 8 Gy at DAY1 after X-ray irradiation. The 4T1 cells were stained with TMRE, and the fluorescence intensity of TMRE was reflected in the mitochondrial activity. After irradiation with 8 Gy, the fluorescence intensity of TMRE decreased under all conditions. The fluorescence intensity of TMRE for the OS/PEI-treated 4T1 cells with or without X-ray irradiation was strong. We performed FCM analyses to evaluate mitochondrial activity (Figure 6C).

Figure 6B illustrates the time-dependence of the mitochondrial activity without NP treatment [NP (-)]. A constant (although, perhaps minimally increased) mitochondrial activity was observed from 0 h to DAY1 and DAY2. Further, a significant decrease in the mitochondrial activity was observed from DAY3 to DAY4, which suggests that the cells were debilitated because they were unable to supply the nutrients necessary for survival under long-term incubation conditions.

The order of mitochondrial activity was higher for OS/PEI, NP (-), and OS/Au/PEI at 0 Gy, 8 Gy, and at all time points (Figure 6C). At 0 h and DAY1 after X-ray irradiation, we compared the mitochondrial activity after treatment with NP (-), OS/PEI, and OS/Au/PEI at 0 Gy. The mitochondrial activity of the cells treated with OS/Au/PEI was weaker than that of cells treated with NP (-). Further, mitochondrial activity of the cells treated with OS/PEI exhibited a slight upward trend in mitochondrial activity compared with that of NP (-). For the cells in DAY1, the mitochondrial activity of OS/Au/PEI was significantly weaker than that of OS/PEI. Further, the 8 Gy X-ray irradiation decreased the mitochondrial activity in NP (-), OS/PEI, and OS/Au/PEI. At DAY4, the mitochondrial activity markedly decreased in all cells. The mitochondrial activity after treatment with OS/Au/PEI with 8 Gy X-ray irradiation was lower than that of OS/PEI; however, the difference in the mitochondrial activity between the treated and untreated cells was small.

The mitochondrial damage levels for OS/Au/PEI may not be sufficient to induce cell death without X-ray irradiation; the viability of the 4T1 cells treated with OS/Au/PEI did not decrease (Figure 3). In addition, the OS/PEI-treated 4T1 cells did not exhibit X-ray sensitizer ability for the NP dose-dependent decrease in cell viability (Figure 3). These results indicate that OS/Au/PEI showed a synergistic effect by the combined damage to DNA and mitochondria.

### 2.7 Non-Apoptotic Cell Death Increased by OS/Au/PEI With X-Ray Irradiation

**FIGURE 7:**
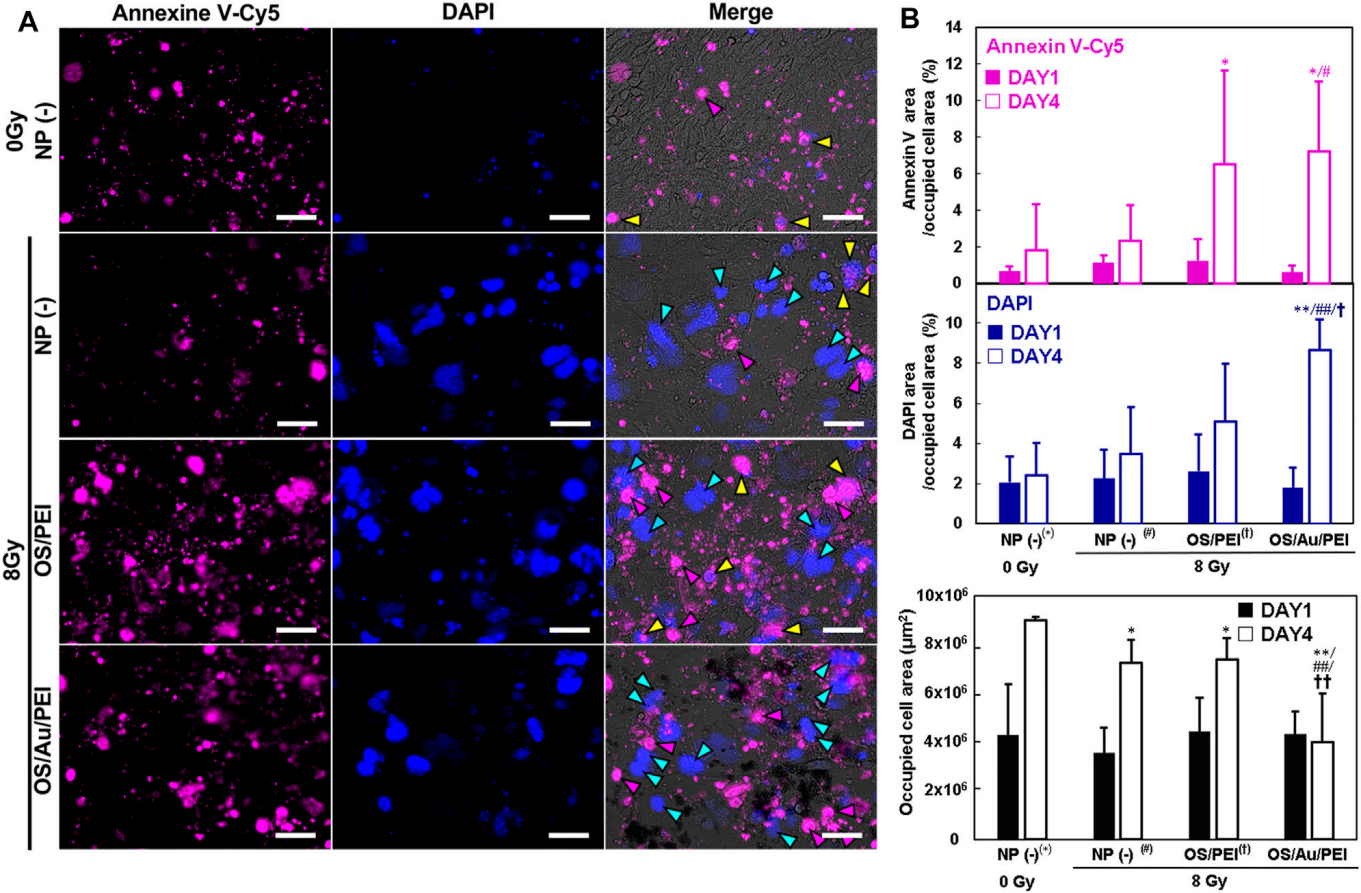
**(A)** FM image of 4T1 cells for apoptotic and necrotic cell determination by Annexin V/DAPI staining treated with 30 μg ml^−1^ of NP (-), OS, OS/PEI, and OS/Au/PEI with X-ray irradiation after DAY4. Scale bar = 50 μm. **(B)** The FM image analysis of 4T1 cells for apoptotic and necrotic cell determination by Annexin V/DAPI staining treated with 30 μg ml^−1^ of NP (-), OS, OS/PEI, and OS/Au/PEI with X-ray irradiation after DAY1 and DAY4. * Significantly different from the 4T1 cells treated with 0 Gy NP (-). # Significantly different from the 4T1 cells treated with 8 Gy NP (-). † Significantly different from the 4T1 cells treated with 8 Gy OS/PEI.

Figure 7A shows the FM images of the 4T1 cells for apoptotic and necrotic cells by annexin V-Cy5/DAPI staining on DAY4. The cells were classified as apoptotic or necrotic by staining with annexin V-Cy5 and DAPI, respectively. Among the annexin V-positive apoptotic cells, the early apoptotic cells did not stain with DAPI, whereas the late apoptotic cells were DAPI-positive. The necrotic cells were DAPI-positive but negative for annexin V-Cy5. Few annexin V-Cy5 positive cells (magenta arrow; early apoptosis) were observed for the untreated cells treated with 0 Gy as the negative control. In addition, some annexin V-Cy5 positive cells were stained with DAPI (yellow arrows; late apoptosis). Early and late apoptotic cell deaths were observed under these conditions. In addition, the morphology shrunk, which was a good assignment to stereotypical morphological changes in the cellular architecture of apoptosis. DAPI-positive and annexin V-Cy5 negative cells (cyan arrow; necrosis) were observed for cells with 8 Gy X-ray irradiation; this morphology was flat and expansive, and the nuclei were large. The morphology was similar to that of senescent cells, which flattened and appeared enlarged, developed cytoplasmic vacuolization, and underwent large-scale chromatin remodeling. (Cho et al., 2004). IR can induce cellular senescence, which is a state of prolonged growth arrest with a permanent loss of proliferative potential. Figure 7B shows the evaluated annexin V-Cy5 and DAPI staining areas, which were divided by the occupied cell area; this indicated the late expression of annexin V-Cy5 and DAPI in all 4T1 cells. At DAY1, there was no considerable difference between annexin V-Cy5 and DAPI, and they occupied all the cell areas. At DAY4, the annexin V-Cy5 area for OS/PEI and OS/Au/PEI with 8 Gy increased significantly for NP (-) treated with 0 Gy. For OS/Au/PEI, it increased significantly for NP (-) at 8 Gy. Further, the DAPI area for OS/Au/PEI increased significantly for NP (-) treated with 0 Gy, and for NP (-) and OS/PEI-treated with 8 Gy. Late apoptotic cells were seldom observed in the FM images; therefore, the significant increase in the DAPI area was caused by an increase in the non-apoptotic cell death. Non-apoptotic cell death for OS/Au/PEI-treated 4T1 cells was promoted, but apoptotic cell death did not promote sufficiently to compare with OS/PEI-treated 4T1 cells by X-ray irradiation. This increase of non-apoptotic cell death is similar to the death due to mitochondrial damage by oxidative stress for the small Au NPs; (Pan et al., 2009); the contribution of mitochondria is required for the induction of apoptosis upon X-ray irradiation. (Daugas et al., 2000; Kang and Reynolds 2009). A previous report showed that mitochondrial deactivation by mitochondrial inhibitors reduces the demand for oxygen by the tumor cells, leading to increased oxygenation and radiation response. (Benej et al., 2018). The mitochondrial activity for the 4T1 cells treated with OS/Au/PEI decreased with or without X-irradiation (Figure 6C). Therefore, it is suggested that OS/Au/PEI has an X-ray sensitizing effect because of the Z-metal theory of Au, and an increase in X-ray sensitizing activity because of the deactivation of mitochondria.

## 3 Conclusion

We demonstrated the X-ray sensitizing potential of OS/Au/PEI in 4T1 cells. The number of OS/Au/PEI particles taken up per 4T1 cell was precisely evaluated using ICP-OES. The number of OS/PEI and OS/Au/PEI was similar, however, the 4T1 cells treated with OS/Au/PEI decreased cell viability and inhibited cell proliferation upon X-ray irradiation compared with that exhibited by OS/PEI (without Au NPs). The OS/Au/PEI-treated 4T1 cells exhibited increased DNA damage attributed to X-ray irradiation and the deactivation of mitochondrial activity; this occurred even without X-ray irradiation. The OS/Au/PEI would affect DNA damage by X-ray irradiation as an X-ray sensitizer and by mitochondrial deactivation as an oxidant. The synergistic effect increased non-apoptotic cell death which was achieved by the combination of OS and the small size of the Au NPs in the application of radiotherapy. Furthermore, the imaging assay of the occupied nucleus area was slightly lower selectivity but revealed to observe the characterized nucleus shape of the structure. The low selectivity was overcome by the combination of the WST-1 assay. We believe the functionalization of Au NPs on OS would gain greater use as an X-ray sensitizer model to application study.

## 4 Experimental Section

### 4.1 Chemicals and Reagents

3-Mercaptopropyltrimethoxysilane (MPMS), 3-aminopropyltrimethoxysilane (APS), fluorescein isothiocyanate isomer (FITC), rhodamine B, bisbenzimide H 33342 trihydrochloride (Hoechst 33342), and the branched polyethyleneimine solution (MW = 2.0k) were purchased from Sigma-Aldrich Chemical Co. (St. Louis, MO, United States). Ammonium hydroxide (NH_4_OH, 28%) and ethanol were purchased from Kishida Chemicals (Osaka, Japan). All other reagents and solvents were of analytical grade.

### 4.2 Synthesis of OS and OS/Au NPs

OS was prepared using a one-pot procedure described previously.(Nakamura et al., 2015). OS/Au was synthesized using the following procedure: First, 1 ml of 10 mg mL^−1^ OS was added to 0.8 ml of 50 mM HAuCl_4_ (aq.) and incubated at 37 °C for 3 days with stirring. Second, the mixture was added to 14.9 ml of water, 1.5 ml of 50 mM sodium citrate, and 0.6 ml of 200 mM NaBH_4_. Third, the solution was incubated at 37°C for 3 days with stirring. Fourth, the mixture was centrifuged (20,000 × g, 30 min) and OS/Au was purified by washing three times with distilled water (DW) to remove excess residues.

### 4.3 Surface Functionalization of OS With Polyethylenimine

OS or OS/Au (1 mg mL^−1^) was allowed to react with 10% PEI2.0k in DW and incubated with mixing for 24 h at 37°C. After incubation, the mixture was centrifuged (20,000 × g, 4°C, 20 min) to remove the unbound PEI; the pellet was washed three times with DW.

### 4.4 Electron Microscopic Observation

The OS and OS/Au were dried and fixed on a 400-mesh copper grid (Nisshin EM Co., Tokyo, Japan) coated with polyvinyl alcohol. STEM and scanning electron microscopy (SEM) images of the NPs were obtained using a Quanta 3D FEG electron microscope (FEI, Hillsboro, OR, United States).

### 4.5 Thermogravimetric Analysis

The thermogravimetric analysis of the NPs was performed using a STA7200 thermal analysis system (Hitachi, Tokyo, Japan) in the temperature range of 30–800°C at a heating rate of 10°C min^−1^ under a 200 ml min^−1^ flow in air. The Au content (w/w (%)) was calculated from the difference in weight loss of OS/Au decomposition and the theoretical weight loss of OS. The theoretical weight loss of OS was assumed from SiO_1.5_C_3_H_6_SH (formula weight: 127.2) to SiO_2_ (formula weight: 60.1) to be 52.8%. The Au content (w/w (%)) was also measured by ICP-OES.

### 4.6 X-Ray Irradiation

X-rays were generated using an X-ray generator ([filter: 0.1 mm Cu and 0.5 mm Al] MBR-1520R-4, Hitachi, Japan). The X-ray dose was controlled automatically by the instruments.

### 4.7 Detection of Hydroxyl Radical

The detection of hydroxyl radicals (•OH) is based on the reaction between aminophenyl fluorescein (APF) and •OH. The assays were performed in black microplates, and the signals were detected using a microplate reader (FlexStation 3, Molecular Devices, United Kingdom). The OS and OS/Au in DW were mixed with APF to achieve a final concentration of 5 µM. The mixtures were excited at 480 nm and fluorescence was detected at 520 nm before and after X-ray irradiation.

### 4.8 Dynamic Light Scattering

The DLS was performed to analyze the size distribution and ζ potential of the NPs using a DelsaMax PRO light scattering analyzer (Beckman Coulter, Brea, CA, United States) at 20°C. The analyte NPs were prepared by dispersing them in DW (0.2 mg ml^−1^).

### 4.9 Evaluation of Cellular Uptake of Nanoparticles by Flow Cytometry

A total of 2.5 × 10^5^ 4T1 cells were plated on 35 mm dishes and incubated at 37 °C for 24 h. The cells were incubated with media containing OS, OS/PEI, OS/Au, or OS/Au/PEI (final concentration: 10 μg ml^−1^) for 24 h. The NPs in the medium were well suspended by sonication before treatment. After incubation, all cells were washed with PBS (−), retrieved from the culture dish, and subjected to flow cytometry. Flow cytometric analysis was performed using aa FACSCalibur™ flow cytometer with Cell Quest software (Becton Dickinson, San Jose, CA, United States) and 488 nm excitation lasers. The fluorescence was detected in the FL1 channel (530/30 nm bandpass filter); a total of 5,000 cells was analyzed for each repetition.

### 4.10 Quantification of Cellular Uptake of NPs by ICP-OES

Cell incubation and sample preparation: For analysis by ICP-OES, a total of 0.5 × 10^6^ 4T1 cells were plated on 10 cm dishes and incubated at 37°C for 24 h. The cells were treated with 10 ml of a medium containing OS, OS/PEI, OS/Au, or OS/Au/PEI (final concentration: 10 μg ml^−1^) for 24 h. The NPs in the medium were well suspended by sonication before treatment. After incubation, all cells were carefully washed three times with PBS (-) to remove excess NPs and dispersed in 2 ml of PBS (-). The concentration (cells ml^−1^) of the dispersed cell samples was determined before the cells were lysed (2.5–3.1 × 10^6^ cells ml^−1^). The samples were centrifuged, and the supernatant was removed after cell counting. The cell pellets were lysed in 2 ml of cell lysis solution.

ICP-OES analysis of cell lysates: ICP-OES (5100 ICP-OES, Agilent Technologies, Mulgrave, Australia) was used to analyze the number of NPs in the cell lysates. Calibration curves were measured using several NPs in the cell lysate solution (2.0 × 10^6^ cells ml^−1^). Before the analysis, the NP-containing cell lysate samples were homogenized by vortexing.

### 4.11 Cell Viability Assay (WST-1 and Imaging Assay of Occupied Nucleus Area)

Cell culture and X-ray irradiation for cell viability assay: 4T1 cells were seeded into 96-well plates at a density of 1,000 cells per well under 100% humidity and cultured at 37°C with 5% CO_2_ for 24 h. Different concentrations (final concentration: 0–100 μg ml^−1^) of NPs (OS, OS/PEI, OS/Au, OS/Au/PEI) were added to the wells. After 24 h, the NPs were irradiated with 8 Gy of X-ray to NPs up-taken 4T1 cells, and the cells were subsequently incubated for 4 days with and without X-ray irradiation.

WST-1 assay: The viability of the 4T1 cells was evaluated using the WST-1 (2-(2-methoxy-4-nitrophenyl)-3-(4-nitrophenyl)-5-(2,4-disulfophenyl)-2H-tetrazolium) cell proliferation assay reagent (Dojin Chemical Co., Kumamoto, Japan). The WST-1 reagent was added 4 days after X-ray irradiation, and the absorbance was measured for each well immediately after the addition of the WST-1 reagent and after incubation at 37°C. The former signal was subtracted from the latter to obtain only the signals derived from the formazan dye (produced by viable cells).

Imaging assay of the occupied nucleus area: The occupied nucleus area was evaluated after the WST-1 assay. The media was removed to evaluate the occupied nucleus area, and it was fixed with 1% paraformaldehyde (PFA) solution and stained with Hoechst33342 after the WST-1 assay. All regions of the well images in the 96-well plate were obtained by FM (BZ-X800, Keyence, Osaka, Japan) using a ×4 objective with stitching. The occupied nucleus area in all wells was analyzed using the Hybrid Cell Count BZ-H4C analyzer software (Keyence, Osaka, Japan).

### 4.12 Evaluation of Cell Count and Cell Viability

A total of 0.4 × 10^5^ 4T1 cells were plated on 35 mm dishes and incubated at 37°C for 24 h. The cells were incubated with a medium containing OS/PEI or OS/Au/PEI (final concentration: 30 μg mL^−1^) for 24 h. The NPs in the medium were well suspended by sonication before treatment. After 24 h, 8 Gy of X-ray was irradiated to NPs up-taken 4T1 cells and incubated at 37°C for the desired time. After incubation, all cells were collected at the desired time; the cells in the supernatant carefully separated by trypsin were collected by centrifugation (1,000 × g, 25°C, 10 min) and re-dispersed in 500 μL of the medium. Automated cell counts were performed using a Vi-CELL^®^ (Beckman, Fullerton, CA, United States). No manual mixing of the samples with trypan blue was required because it was performed automatically by the instrument.

### 4.13 Evaluation of Mitochondrial Activity

Cell culture and X-ray irradiation were performed to evaluate cell counts and viability. All cells were collected after incubation. The cells in the supernatant were carefully separated by trypsin, collected by centrifugation (1,000 × g, 25°C, 10 min), re-dispersed, stained with tetramethylrhodamine ethyl ester perchlorate (TMRE, Invitrogen, Eugene, OR, United States), and subjected to flow cytometric analysis. Flow cytometric analysis was performed using a FACSCaliburTM flow cytometer with Cell Quest (Becton Dickinson, San Jose, CA, United States) and 488 nm excitation lasers. A total of 5,000 cells were analyzed for each repeat. Fluorescence was detected on the FL2 channel (filter with a passband of 543–627 nm).

### 4.14 Imaging Assay (DNA Double-Strand Breaks and Annexin V/DAPI Assay)

Cell culture and X-ray irradiation for imaging assays (DNA DSBs and Annexin V/DAPI assay): The 4T1 cells were seeded in a 96-well plate and incubated at 37°C for 24 h. Thereafter, the NPs (OS/PEI and OS/Au/PEI) were added to the 96-well plate at a final concentration of 30 μg ml^−1^. After 24 h, 8 Gy of X-ray was irradiated to NPs up-taken 4T1 cells, which were incubated at 37°C for the desired time.

DNA double-strand breaks: The DNA DSBs were measured by the immunofluorescence staining of γ-H2AX to evaluate the DNA damage caused by X-ray irradiation. The immunofluorescence staining of γ-H2AX was performed using a DNA damage detection kit (Dojin Chemical Co., Kumamoto, Japan) after 1 or 6 h of X-ray irradiation. DAPI was used to stain the cell nuclei. The images were obtained in 3 × 3 stitching areas at a 10× magnification, which were selected in the central part of the well and contained more than 1,000 cells by BZ-X800 (Keyence, Osaka, Japan). The quantification was performed using Hybrid Cell Count BZ analyzer software (Keyence, Osaka, Japan). The percentage of stained γ-H2AX expression cells per cell was calculated.

Annexin V-Cy5/DAPI assay: At the desired time, the cells were stained using the Annexin V-Cy5 apoptosis detection kit (Bio-Vision, Inc. Milpitas, CA, United States), followed by DAPI staining. Images were obtained in 3 × 3 stitching areas at 10 × magnification, which were selected in the central part of the well and contained more than 1,000 cells by BZ-X800 (Keyence, Osaka, Japan). Quantification was performed using the Hybrid Cell Count BZ analyzer software (Keyence, Osaka, Japan). The percentage of the stained area of annexin V-Cy5 and DAPI per cell per nucleus was calculated. Thereafter, the area of Annexin V-Cy5 and DAPI per cell was calculated as [area of Annexin V-Cy5 or DAPI]/[area of cell by bright field images (μm^2^)].

### 4.15 Statistical Analysis

The data are presented as mean ± standard deviation from three replicates and analyzed by one-way analysis of variance (ANOVA) followed by the Tukey–Kramer test in Microsoft Excel; statistical significance was set at *p < 0.05 and **p < 0.01.

## Data Availability

The original contributions presented in the study are included in the article/Supplementary Material, further inquiries can be directed to the corresponding author.

## Abbreviations

NPs, Nanoparticles; OS, Organosilica NPs; OS/Au, Organosilica NPs with Au NPs; PEI, Polyethyleneimine; IR, Ionizing radiation; ROS, Reactive oxygen species; •OH, Hydroxyl radicals; EPR, Enhanced permeability and retention; DDS, Drug delivery system; WST-1, Water soluble tetrazolium salts; MTT, 3-(4,5-dimethylthiazol-2-yl)-2,5-diphenyl-2H-tetrazolium bromide; 4T1, Mouse mammary tumor cell line; FITC, Fluorescein isothiocyanate; STEM, Scanning transmission electron microscopy; APF, Aminophenyl fluorescein; SPR, Surface plasmon resonance; FM, Fluorescence microscopy; DLS, Dynamic light scattering; FCM, Flow cytometry; ICP-OES, Inductively coupled plasma optical emission spectroscopy; DNS DSBs, DNA double-strand breaks; TMRE, Tetramethylrhodamine methyl ester.
