# Supplementary material for: Surface Functionalization of Organosilica Nanoparticles With Au Nanoparticles Inhibits Cell Proliferation and Induces Cell Death in 4T1 Mouse Mammary Tumor Cells for DNA and Mitochondrial-Synergized Damage in Radiotherapy
Source: Front Chem. 2022 May 10;10:907642. doi: 10.3389/fchem.2022.907642 (PMC9127317; doi:10.3389/fchem.2022.907642)
Supplement: Supplementary file 1 [file Table1.DOCX]

**Supplementary Information**

**Surface Functionalization of Organosilica Nanoparticles with Au Nanoparticles inhibits Cell Proliferation and Induces Cell Death in 4T1 Mouse Mammary Tumor Cells for DNA and Mitochondrial-synergized Damage in Radiotherapy**

*Chihiro Mochizuki, Yukihito Kayabe, Junna Nakamura, Masaya Igase, Takuya Mizuno, Michihiro Nakamura^*^*


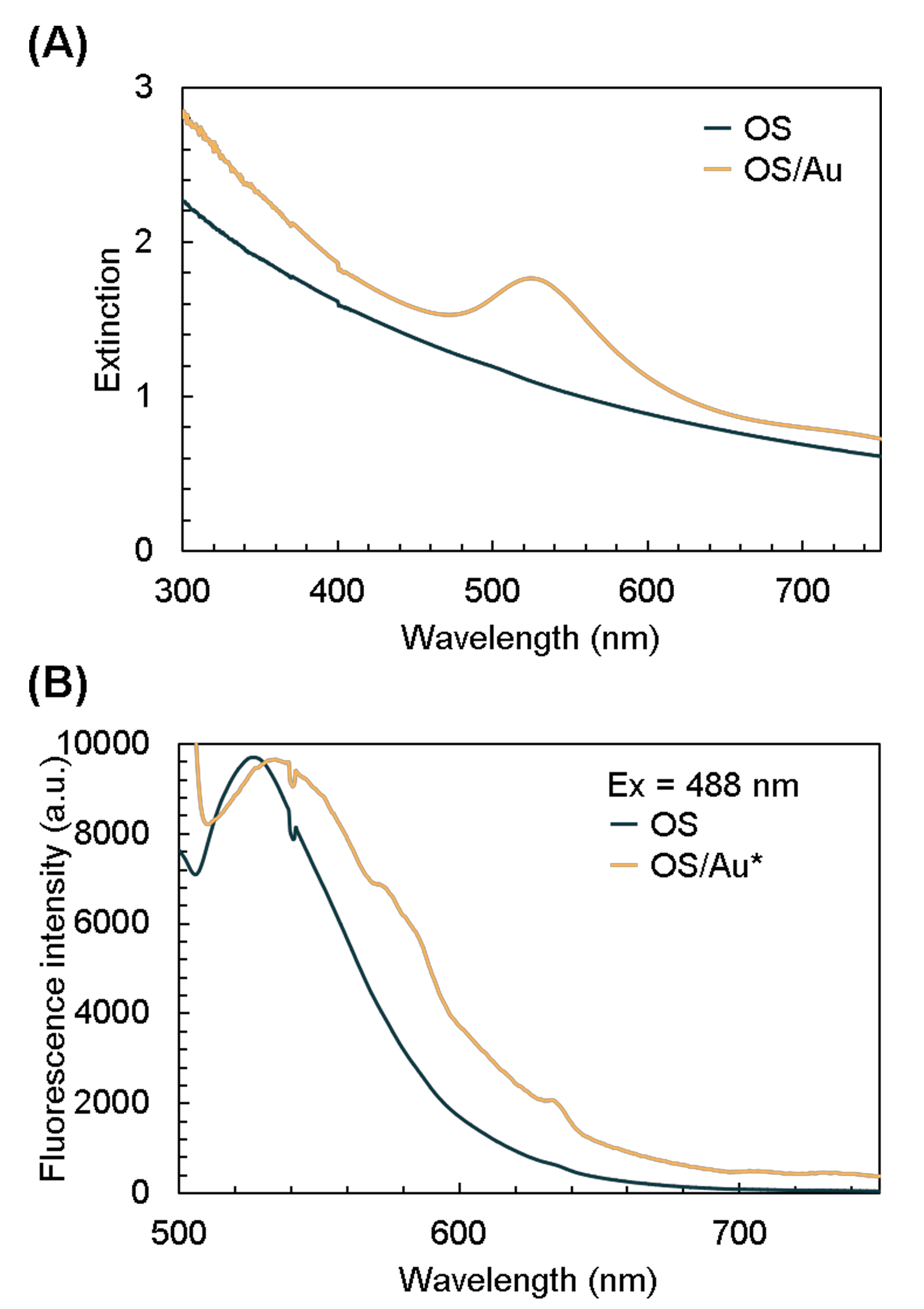


**Figure S1.** Extinction spectra (A) and fluorescence spectra (B) of OS and OS/Au.*The spectra were magnified 26 times.


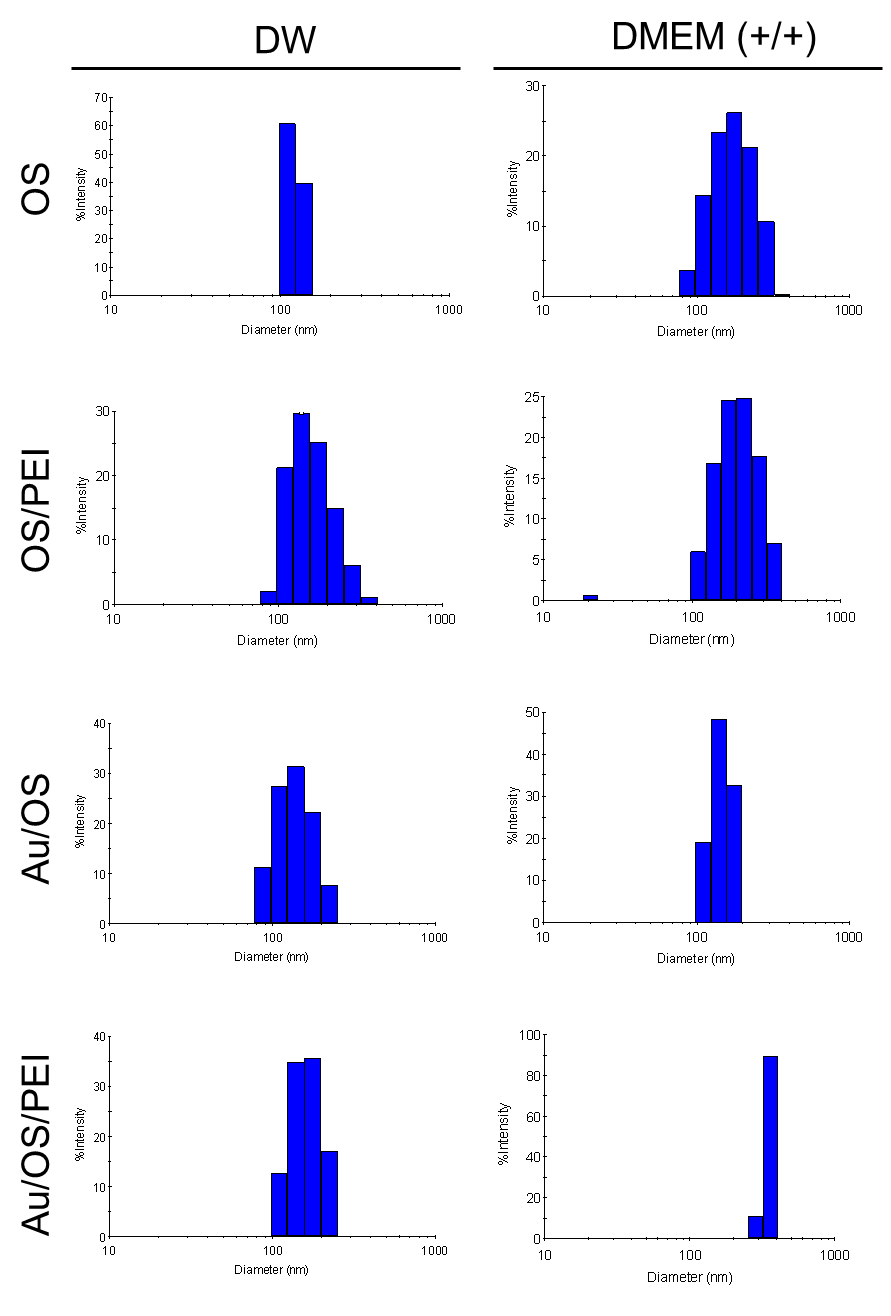


**Figure S2.** The distribution of diameter by DLS.
